# Phytochemical Screening of 45‐Million‐Year‐Old Colored Angiosperm Leaves Reveals Distinctive Chlorophyll‐Derived and Polyphenolic Pigments

**DOI:** 10.1111/gbi.70042

**Published:** 2026-01-31

**Authors:** Klaus Wolkenstein, Christa E. Müller, Marianne Engeser, Holm Frauendorf, Victoria E. McCoy, Carole T. Gee

**Affiliations:** ^1^ Pharmaceutical Institute, Pharmaceutical & Medicinal Chemistry University of Bonn Bonn Germany; ^2^ Department of Geobiology, Geoscience Centre University of Göttingen Göttingen Germany; ^3^ Kekulé Institute for Organic Chemistry and Biochemistry University of Bonn Bonn Germany; ^4^ Institute of Organic and Biomolecular Chemistry University of Göttingen Göttingen Germany; ^5^ Department of Geosciences University of Wisconsin–Milwaukee Milwaukee Wisconsin USA; ^6^ Bonn Institute of Organismic Biology, Division of Paleontology University of Bonn Bonn Germany

**Keywords:** chlorophyll, cyclopheophorbide enol, exceptional preservation, flavonoids, fossil leaves, mass spectrometry, pigments

## Abstract

Present‐day angiosperm plants produce a plethora of metabolites including pigments that serve for important functions such as photosynthesis, protection against light, attraction of pollinators, and defense against microbes and herbivores. However, little is known about phytochemical constituents of ancient angiosperms, their distribution in the fossil record, their stability in deep time, and diagenesis. Outstanding preservation of ancient angiosperms, including exceptional color preservation, has been reported, but chemical analyses of such valuable specimens are limited by the rarity of the fossil material and the small amounts of potentially preserved metabolites. Here we use highly sensitive targeted liquid chromatography–tandem mass spectrometry in multiple reaction monitoring mode to screen for nanogram quantities of intact ancient phytochemical metabolites and their products in exceptionally well‐preserved, about 45‐Ma‐old leaves from the Eocene Geiseltal fossil Lagerstätte, Germany. We show that diverse chlorophyll derivatives and degradation products as well as polyphenolic pigments are preserved in green to yellow colored angiosperm leaves and the brown coal matrix from Geiseltal. Most interesting is the fossil occurrence of the “unstable” green chlorophyll derivative dihydro‐13^2^,17^3^‐cyclopheophorbide *a*‐enol, since cyclopheophorbide‐enols are otherwise known as unique non‐fluorescent chlorophyll catabolites of microorganisms in modern aquatic environments. The monopyrrole hematinic acid is interpreted as a stable product of chlorophyll catabolism via linear tetrapyrroles. Moreover, polyphenolic compounds in the fossil angiosperms are represented by the flavonoid pigments apigenin and luteolin. Our results demonstrate the potential of paleometabolomic‐like screening of individual plant fossils to trace the fate of phytochemical constituents and to understand the processes of fossilization at the molecular level.

## 1 Introduction

Exceptionally well‐preserved fossil leaves that still show green color have been reported from the Eocene brown coals (lignites) of Geiseltal, Germany (Weigelt and Noack 1932; Rüffle 1976). Such outstanding color preservation is a phenomenon that has only been observed at very few additional localities worldwide including Succor Creek (Miocene, Oregon, USA) (Niklas and Giannasi 1977a), Clarkia (Miocene, Idaho, USA) (Smiley and Rember 1985), and Willershausen (Pliocene, Germany) (Wolkenstein and Arp 2021). Because the preservation of biological pigments in fossil leaves is still poorly studied (Gee and McCoy 2021; Locatelli 2014), and major advances in analytical chemical techniques have been achieved since previous investigations several decades ago (Dilcher et al. 1970), we aimed to analyze the metabolomic profile of colored fossil leaves from Geiseltal in detail using state‐of‐the‐art methods with a focus on organic pigments and their degradation products.

**FIGURE 1:**
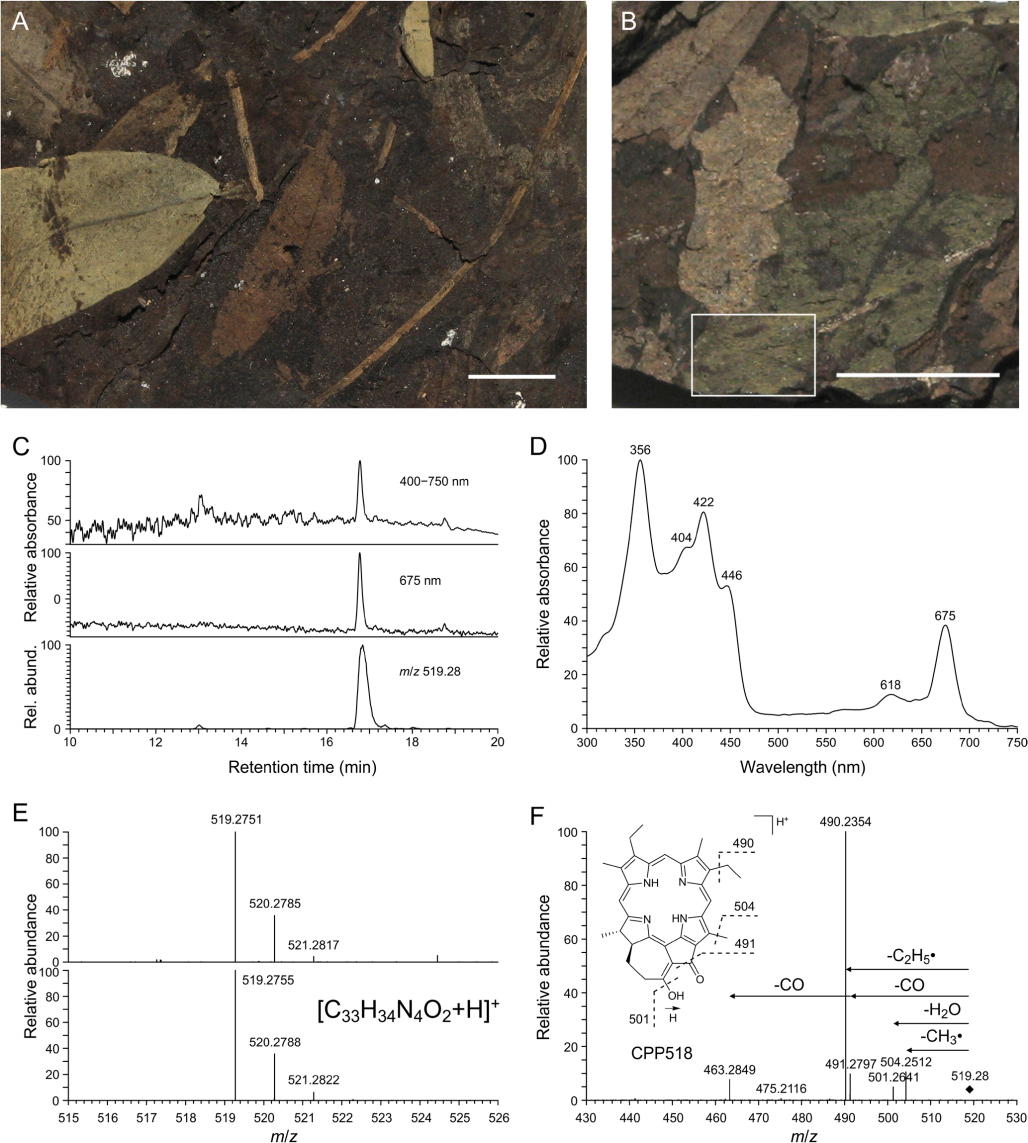
Examples of colored dicotyledon leaves from the Eocene of Geiseltal, Germany, and analytical data (HPLC‐DAD‐HRMS) of the fossil chlorophyll derivative dihydro‐13^2^,17^3^‐cyclopheophorbide *a*‐enol (CPP518) extracted from green colored leaf specimen. (A) Lignite slab with green, yellowish, and brown colored leaves (MB.Pb.2023.0100). (B) Green colored leaf (MB.Pb.2023/0087.1) with indicated sample area (G1a). (A and B) scale bar, 1 cm. (C) HPLC chromatograms (absorbance at 400–750 nm and 675 nm) and corresponding extracted ion chromatogram (*m*/*z* 519.28, positive‐ion ESI‐HRMS) of acetone extract (G1a) using elution gradient 1. (D) UV–visible (DAD) spectrum of peak shown in C. (E) Comparison of the mass spectrum of the peak in the extracted ion chromatogram shown in C (Upper) to the simulated mass spectrum for [C_33_H_34_N_4_O_2_ + H]^+^ (Lower). (F) Collision‐induced fragmentation of the *m*/*z* 519.28 ion peak in the extracted ion chromatogram and proposed fragmentation of the [M + H]^+^ ion of CPP518.

The Geiseltal fossil Lagerstätte is well known for exceptional preservation of plant and animal fossils and has yielded fossil remains including articulated skeletons and details of soft tissues (Krumbiegel et al. 1983). Furthermore, organic preservation of fossilized latex (“monkeyhair”) (McCoy et al. 2021) and resins (Simoneit et al. 2021) has been described. As mentioned in previous reports, even at Geiseltal only a small part of the leaves show a distinct green coloration (Dilcher et al. 1970), while other leaves are rather yellowish and the majority of specimens show various shades of brown (Figures 1A,B and S1). In some cases, a whitish layer of leaves can be observed (Malekhosseini et al. 2025). Fossil angiosperm leaves with green coloration were mainly found at Geiseltal within laminated lignites deposited in shallow pits (“Trichter”), which have been interpreted as synsedimentary sinkhole structures (Krumbiegel 1975). The exceptional preservation of fossils in these depressions has been explained by an anaerobic environment, influenced by alkaline karstic water, which neutralized humic acids from the peat/brown coal (Krumbiegel 1975; Weigelt and Noack 1932).

Bulk analyses of lignite samples from Geiseltal containing green leaves were performed as early as 1932 (Weigelt and Noack 1932). These analyses based on early spectrophotometry of extracts revealed that green pigments related to chlorophyll are preserved. In 1967, Dilcher (1967) performed more advanced bulk analyses, using paper chromatography and UV–visible spectroscopy, and tentatively identified pheophytin *a* as a major chlorophyll derivative. However, based on further experiments including electron ionization mass spectrometry (Dilcher et al. 1970), it was possible to exclude pheophytin *a* and to determine the chlorophyll derivative as methyl pheophorbide *a* (Figure S2).

Currently, high‐performance liquid chromatography coupled with mass spectrometry (HPLC‐MS) is regarded as a method of choice for screening of non‐volatile metabolites such as pigments in present‐day organisms. However, although several studies have been conducted on chlorophyll‐derived pigments in ancient sediments using HPLC‐MS (Mawson et al. 2004; Mawson and Keely 2008; Junium et al. 2011; Woltering et al. 2016), only very few studies have been published using this methodology for the analysis of metabolites from specific fossil plants. Polyphenolic pigments of Cretaceous *Ginkgo* leaves (Zhao et al. 2006) and Jurassic calcareous red algae (Wolkenstein et al. 2010) have been reported so far, as well as suberin from the bark of the Geiseltal “monkeyhair” tree (Tahoun et al. 2024). One reason for this limited use is that such analyses require sampling of fossil material in sufficient quantities that allow for the detection of the usually tiny amounts of analytes within a complex matrix. The invention of tandem mass spectrometry (MS/MS) has significantly increased the detection sensitivity, and the development of advanced and rapid mass spectrometry scanning techniques such as multiple reaction monitoring (MRM) has allowed for the continuous measurement of specific fragment ions of multiple analytes coupled with on‐line chromatography (Kitteringham et al. 2009).

Here, we use HPLC coupled with highly sensitive and selective targeted MS/MS in MRM mode for the analysis of fossil leaves from Geiseltal in order to: (i) detect even trace amounts of preserved metabolites while using as little as possible from the valuable fossil material for destructive analysis, (ii) obtain quantitative data of plant metabolites of individual leaves, and (iii) explore the potential of metabolomic studies of fossil plants. Extracts of individual Geiseltal leaves were screened by targeted HPLC‐MS/MS analysis for 30 common plant metabolites and known or suspected degradation products including chlorophyll derivatives, polyphenolic pigments (flavonoids), and carotenoids within a single run (Table S1). Because in fossil samples many unknown degradation products are to be expected in addition to preserved metabolites, major non‐targeted compounds were characterized and identified by HPLC with diode array detection coupled with high‐resolution mass spectrometry (HPLC‐DAD‐HRMS).

## 2 Materials and Methods

### 2.1 Samples and Reference Compounds

Compression fossils of several individual leaves of dicotyledons with colors ranging from distinct green to yellowish to brown from the Middle Eocene of Geiseltal near Halle/Saale, eastern Germany were selected from collection material of the Museum of Natural History Berlin (MB) (Table S2). The fossil material was collected in 1970 from the “Mittelkohle” of pit (“Trichter”) 35 (with exception of MB.Pb.2023/0087.1 collected in pit 37) within the Neumark‐Süd area of the Geiseltal brown coal open‐cast mine. Today, the Geiseltal outcrop is flooded and the leaf‐bearing sediments are no longer accessible. For comparison, present‐day leaves of *Fagus sylvatica* were collected: a fresh green leaf and a green leaf that was collected and freeze‐dried about 10 years ago and was stored at room temperature in the dark.

For reference, the following standard compounds from the compound library of the Pharmaceutical Institute of the University of Bonn were used: (i) chlorophylls and their derivatives: chlorophyll *a*, pheophytin *a*, pheophorbide *a*, methyl pheophorbide *a*, (ii) monopyrrolic compounds: hematinic acid, (iii) phenolic acids: *p*‐coumaric acid, (iv) flavonoids: flavones: apigenin, luteolin; isoflavones: genistein; flavonols: kaempferol, quercetin; flavanonols: dihydrokaempferol; flavone glycosides: apigenin‐8*C*‐glucoside (vitexin); flavonol glycosides: kaempferol‐3*O*‐glucoside, quercetin‐3*O*‐glucoside, quercetin‐3*O*‐galactoside (hyperoside), quercetin‐3*O*‐rhamnoside, quercetin‐3*O*‐rutinoside (rutin), (v) carotenoids: lutein, zeaxanthin, β‐carotene. Hematinic acid was isolated as described previously (Tahoun et al. 2023).

### 2.2 Sample Preparation and Extraction

Small parts of individual fossil leaves (less than 1 cm^2^ leaf surface) were cut or scraped off from the sediment matrix (3–128 mg). Additional samples of the sediment matrix (lignite) adjacent to two leaves that contained almost no visible leaf fragments were collected as well (13 and 77 mg). As with the fossil material, only small parts of the present‐day leaves were used as samples (13 mg of freeze‐dried and 25 mg of fresh leaf). Samples were ground with a glass rod and extracted (2×) by sonication (10 min at 40°C) with argon‐purged acetone/methanol 1:1 (vol/vol), followed by extraction with argon‐purged acetone (1×). Only glass surfaces were used until analysis to exclude contaminations from plastics, and brown glass was used in order to protect extracts from light. Aliquots of all extracts were centrifuged before further analysis.

### 2.3 Targeted HPLC‐MS/MS Analysis

All extracts were analyzed directly following sample preparation by targeted HPLC‐MS/MS in multiple reaction monitoring (MRM) mode on an Agilent 1290 Infinity II liquid chromatography system coupled to a Sciex QTRAP 6500+ hybrid triple quadrupole‐linear ion trap mass spectrometer equipped with an electrospray ionization (ESI) source. Separation was performed at 50°C on an Agilent Zorbax SB C18 column (50 × 2.1 mm, 1.8 μm). Eluent A was water with 0.1% formic acid and eluent B was acetonitrile/2‐propanol 3:7 (vol/vol) with 0.1% formic acid. The HPLC program consisted of a linear gradient (elution gradient 1) of 5% B to 100% B in 20 min, followed by isocratic elution at 100% B for 5 min at a flow rate of 0.4 mL min^−1^. The injection volume was 2 μL. MRM transitions (Table S1) were acquired in positive‐ and negative‐ion mode (ion spray voltage 4500 V and −4500 V, 500°C). MRM parameters were optimized by direct infusion using solutions of standard compounds, and MRM transitions of additional flavonoid glycosides were derived based on the MRM of used standard compounds. MRM transitions of fossil compounds found by non‐targeted HPLC‐DAD‐HRMS (see below) were determined and optimized using a leaf extract from Geiseltal.

To exclude any cross‐contamination of samples, a procedural blank was measured before the fossil extracts, and fossil extracts were measured in duplicates followed by measurement of standard compounds. Compounds were identified by comparison of retention time, MRM transitions, and proportion of MRM transitions to those of standard compounds. For confirmation of detected fossil analytes, additional HPLC‐MS/MS analyses were carried out using a different chromatographic system (elution gradient 2) with pure methanol as eluent B (instead of acetonitrile/2‐propanol 3:7 with 0.1% formic acid) and keeping the other chromatographic conditions. For these analyses an additional (third) MRM transition was measured for each compound, and the number of scanned MRM transitions within one cycle was reduced to those analytes to be confirmed to improve the signal‐to‐noise ratio. The injection volume was 2 or 4 μL.

Quantitative determination was performed using quantifier MRM transitions of leaf extracts (acetone/methanol 1:1) and external standards. For determination of recovery, the residue of an already extracted sample of sediment matrix from Geiseltal (G15M) was spiked with known concentrations of the standard compounds hematinic acid, apigenin, luteolin, methyl pheophorbide *a*, and pheophytin *a*, and was extracted and analyzed as described above.

### 2.4 Non‐Targeted HPLC‐DAD‐HRMS Analysis

HPLC‐DAD‐HRMS measurements were carried out on a Thermo Accela HPLC with a Thermo Finnigan Surveyor photodiode array detector (DAD) coupled to a Thermo LTQ Orbitrap XL with ESI source using the same chromatographic conditions (elution gradient 1) as above. DAD spectra were recorded in the 200–800‐nm wavelength range and were background corrected by subtraction of a 0.15–0.25 min range directly in front of the respective peaks. Mass spectra were acquired in positive‐ion mode over the 250–1500‐*m*/*z* range at a mass resolution of 60,000. Mass calibration was established externally using the LTQ XL ESI Hybrid CalMix Solution. Collision‐induced fragmentation spectra of selected precursor ions were obtained at 40% normalized collision energy (NCE).

## 3 Results

### 3.1 Chlorophyll Derivatives

**FIGURE 2:**
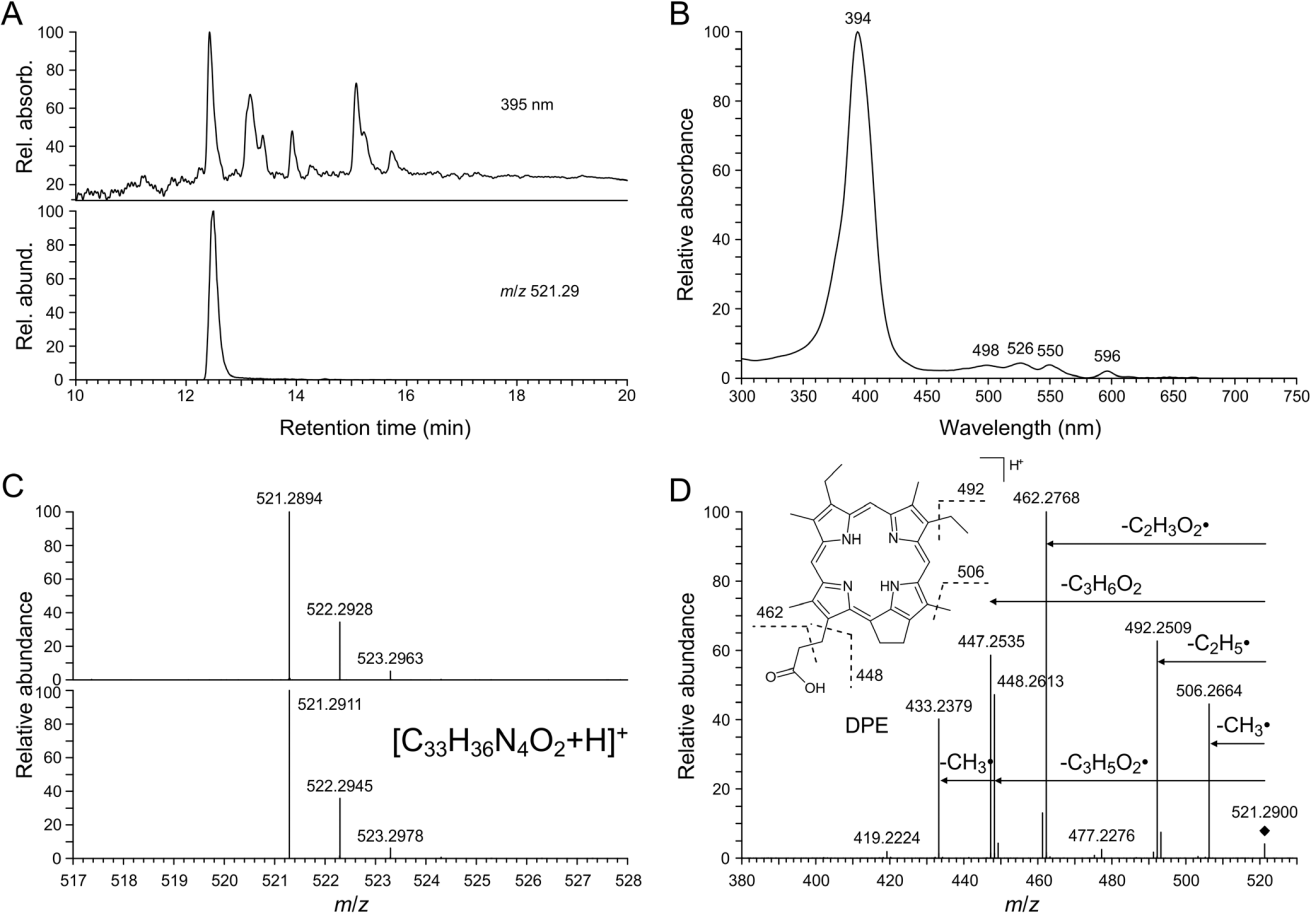
Analytical data of desoxophylloerythrin (DPE) extracted from green Eocene leaf from Geiseltal (G2a acetone/methanol 1:1 extract, elution gradient 1). (A) HPLC chromatogram (absorbance at 395 nm) and corresponding extracted ion chromatogram (*m*/*z* 521.29, positive‐ion ESI‐HRMS). Note missing peak of CPP518 which has already degraded in the acetone/methanol 1:1 extract to (*S*/*R*)‐dihydrochlorophyllone *a* (see Figure S4). (B) UV–visible (DAD) spectrum of peak with retention time 12.5 min shown in A. (C) Comparison of the mass spectrum of the peak in the extracted ion chromatogram shown in A (Upper) to the simulated mass spectrum for [C_33_H_36_N_4_O_2_ + H]^+^ (Lower). (D) Collision‐induced fragmentation of the *m*/*z* 521.29 ion peak in the extracted ion chromatogram and proposed fragmentation of the [M + H]^+^ ion of DPE.

**FIGURE 3:**
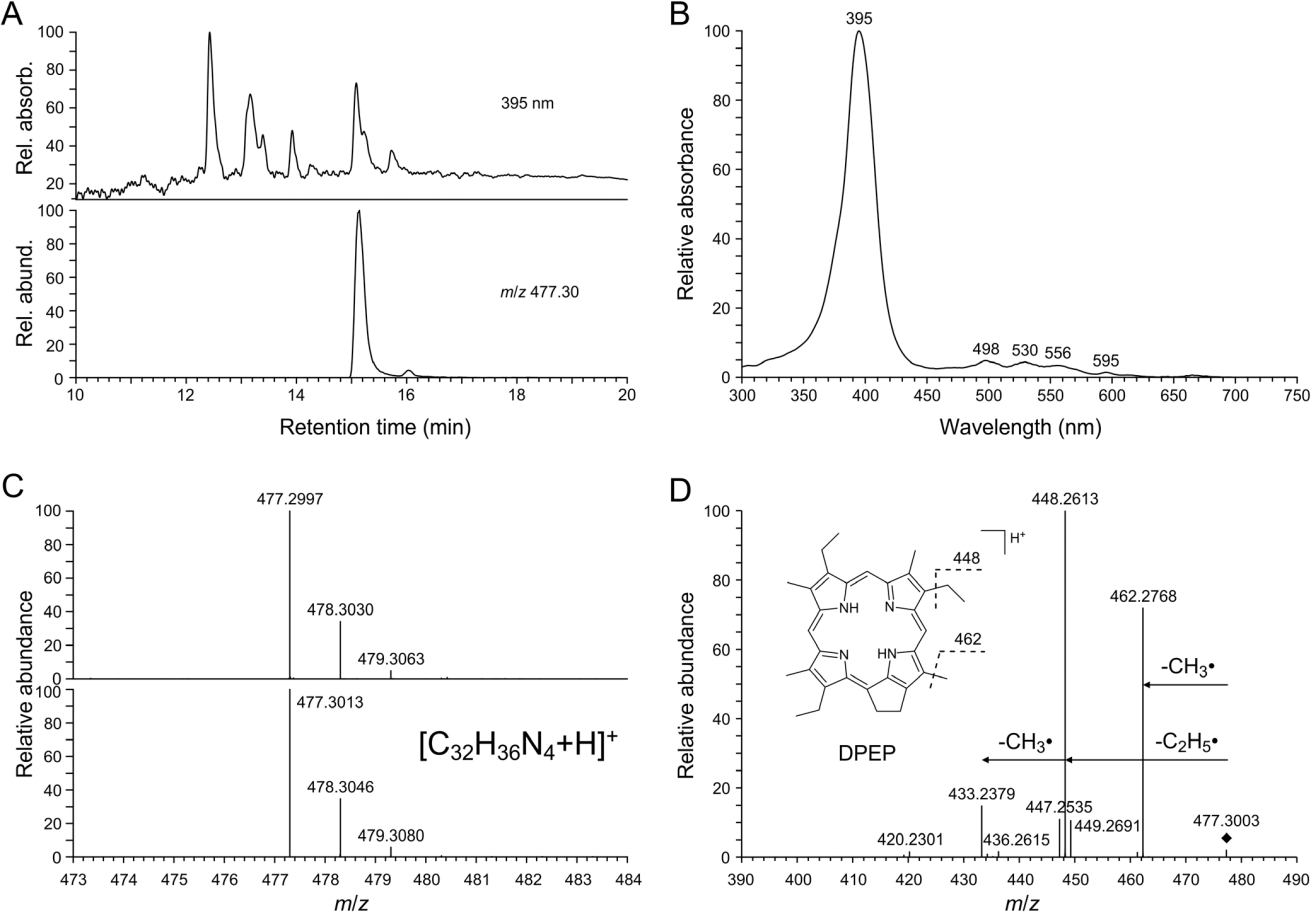
Analytical data of DPEP extracted from green Eocene leaf from Geiseltal (G2a acetone/methanol 1:1 extract, elution gradient 1). (A) HPLC chromatogram (absorbance at 395 nm) and corresponding extracted ion chromatogram (*m*/*z* 477.30, positive‐ion ESI‐HRMS). (B) UV–visible (DAD) spectrum of peak with retention time 15.1 min shown in A. (C) Comparison of the mass spectrum of the peak in the extracted ion chromatogram shown in A (Upper) to the simulated mass spectrum for [C_32_H_36_N_4_ + H]^+^ (Lower). (D) Collision‐induced fragmentation of the *m*/*z* 477.30 ion peak in the extracted ion chromatogram and proposed fragmentation of the [M + H]^+^ ion of DPEP.

Non‐targeted HPLC‐DAD‐HRMS analysis of Geiseltal leaf extracts (acetone/methanol 1:1) revealed several peaks with similar UV–visible spectra showing a distinct *Soret* band (at about 400 nm) (Figures 2 and 3), which is characteristic of porphyrins and chlorophyll derivatives in general (Tahoun et al. 2021; Callot and Ocampo 2000). Based on their UV–visible spectra, accurate mass spectrometry data, and collision‐induced fragmentation in comparison with published data (Mawson et al. 2004), several uncomplexed porphyrin pigments were identified (Figures 2 and 3), the compound with the molecular formula C_33_H_36_N_4_O_2_ was assigned to desoxophylloerythrin (DPE), and the compound with the molecular formula C_32_H_36_N_4_ to desoxophylloerythroetioporphyrin (DPEP). However, with regard to the green color of the leaves, one major compound with a longer retention time, which was also present in acetone extracts, was of particular interest (Figure 1C). Its UV–visible spectrum (Figure 1D) deviated considerably from the others (Figures 2 and 3), showing similarity with those of cyclopheophorbide‐enols, green chlorophyll derivatives with a seven‐membered ring (Falk et al. 1975). A corresponding extracted ion chromatogram (positive ions) showed a distinct signal at *m*/*z* 519 for the compound (Figure 1C). By accurate mass spectrometry, a molecular formula of C_33_H_34_N_4_O_2_ was determined (Figure 1E), requiring 19 degrees of unsaturation. Characteristic fragmentation was obtained by collision‐induced dissociation showing the elimination of C_2_H_5_^•^, CH_3_^•^, CO, 2CO, and H_2_O (Figure 1F). By comparison with published spectroscopic data and retention behavior in reversed‐phase HPLC (Goericke et al. 2000), the compound was identified as dihydro‐13^2^,17^3^‐cyclopheophorbide *a*‐enol (CPP518). Although the stereochemistry of the diastereomeric compound was not investigated in the present study, we believe that the compound has not racemized and retained its original configuration, since only a single peak is observed in the chromatogram. During repeated HPLC‐MS/MS measurements of the same extracts, it was observed that the compound, in contrast to the other chlorophyll derivatives, was not stable in the extraction solvent (acetone/methanol) and showed formation of almost equal amounts of two isomeric degradation products within a few hours (Figure S3), which is also in agreement with published observations of cyclopheophorbide‐enols (Aydin et al. 2003; Ocampo et al. 1999). Based on UV–visible spectroscopy, accurate mass data, and characteristic fragmentation (Figures S3 and S4) in comparison with published data (Mawson and Keely 2008), the observed degradation products of CPP518 were identified as (*S*)‐ and (*R*)‐dihydrochlorophyllone *a*.

**FIGURE 4:**
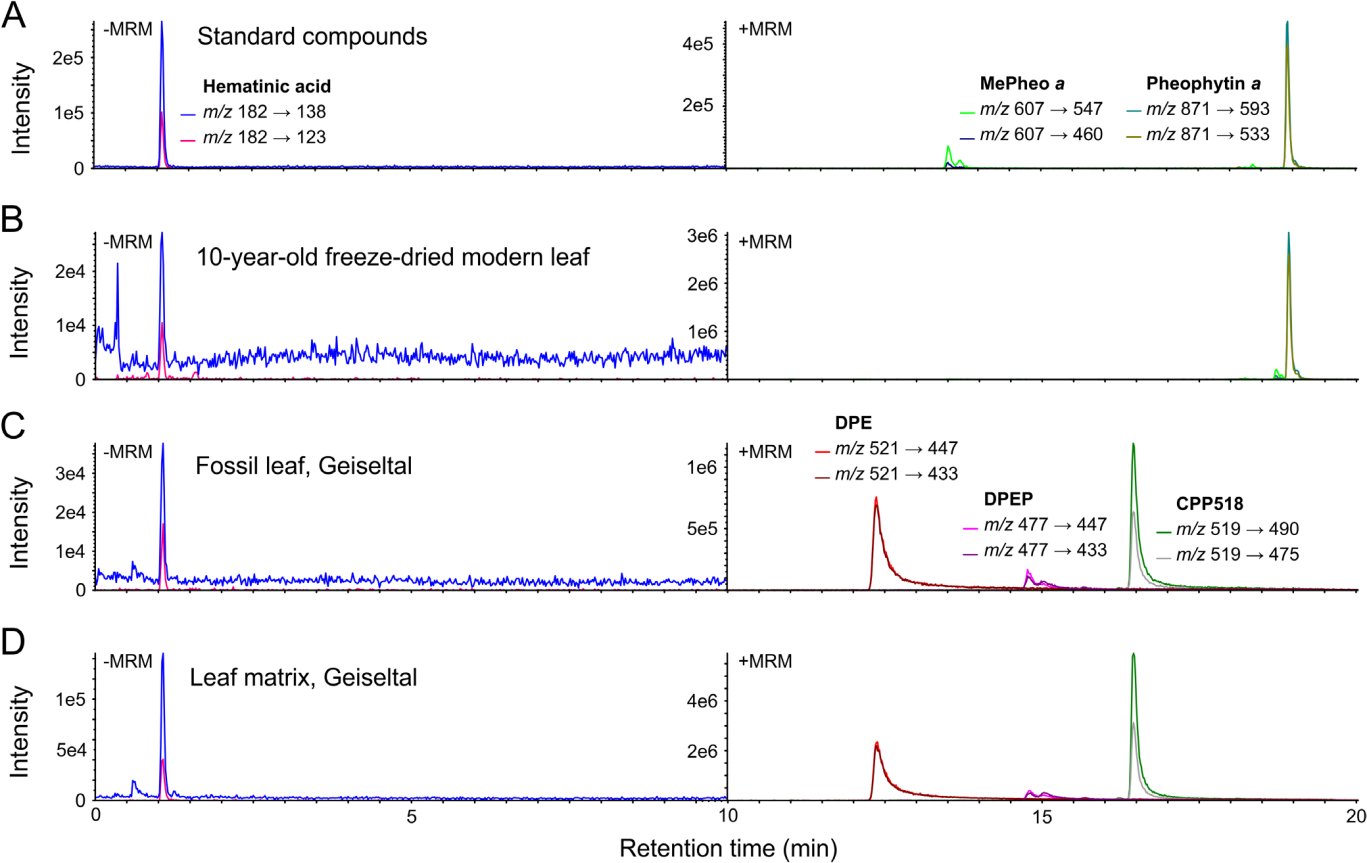
Targeted MRM analysis (elution gradient 1) of chlorophyll degradation products (acetone/methanol 1:1 extracts) of fossil leaf and leaf matrix in comparison to modern leaf and standard compounds. (A) MRM chromatograms of standard compounds. (B) MRM chromatograms of 10‐year‐old freeze‐dried modern *Fagus* leaf. (C) MRM chromatograms of fossil leaf from Geiseltal (G15a). (D) MRM chromatograms of sediment matrix of leaf from Geiseltal (G15M). MRM chromatograms with retention time 0–10 min were recorded in negative‐ion mode and MRM chromatograms with retention time 10–20 min were recorded in positive‐ion mode. Note different intensity scales for negative‐ and positive‐ion MRM, and that quantifier and qualifier ion transitions in MRM were obtained by optimized parameters and therefore do not always correspond to the most intense fragmentation ions of the compounds in the MS/MS spectra obtained by HRMS.

Using HPLC‐MS/MS, the green pigment CPP518 was detected in all green and yellowish fossil leaf samples, but also in a brown fossil leaf and the lignite sediment matrix (Figure 4, Table S2). By contrast, no CPP518 was found in either a fresh or a 10‐year‐old freeze‐dried modern angiosperm leaf (*Fagus*). The red porphyrin DPE was found in all fossil leaf samples as well as in the sediment matrix. Despite the extremely low detection limits (0.05 and 0.01 ng mL^−1^) and the high recovery of standards (93 and 101%), no traces of the chlorophyll derivatives methyl pheophorbide *a* and pheophytin *a*, whose occurrence from Geiseltal has previously been reported (Dilcher 1967; Dilcher et al. 1970), were detected in the fossil leaf samples or the matrix sediment (Figure 4). However, both compounds (main compound pheophytin *a*) were detected in a fresh modern and a 10‐year‐old freeze‐dried *Fagus* leaf.

### 3.2 Monopyrrolic Chlorophyll Degradation Products

By targeted HPLC‐MS/MS, the chlorophyll degradation product hematinic acid was found. The fossil compound showed the same retention time and MRM transitions (same ratio of quantifier to qualifier) as the standard compound (Figure 4). The identity of the compound was confirmed by using a second chromatographic system (Figure S5), showing again the same retention time and MRM transitions for fossil compound and standard. Hematinic acid was also detected in all fossil leaf samples and the lignite sediment matrix with concentrations ranging from 9 to 51 ng mg^−1^ (Table S2). Furthermore, a clear correlation (*R* ^2^ = 0.92) between the relative amounts of hematinic acid and CPP518 in the samples was observed. Hematinic acid was not detected in the fresh modern *Fagus* leaf; however, it was present in the 10‐year‐old freeze‐dried *Fagus* leaf (Figure 4).

### 3.3 Polyphenolic Pigments

**FIGURE 5:**
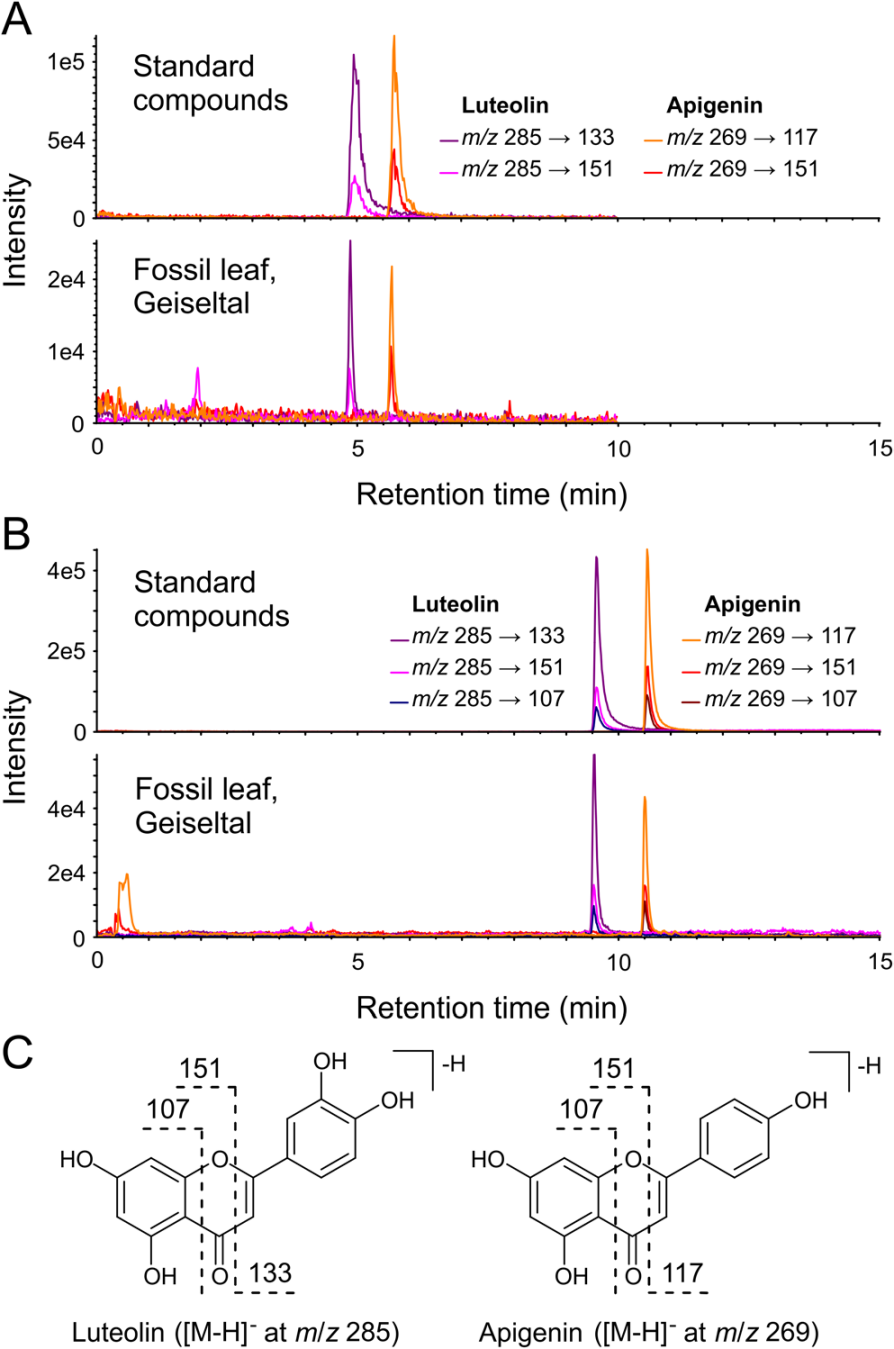
Targeted MRM analysis (negative‐ion mode) of flavonoid pigments (acetone/methanol 1:1 extract) of Geiseltal leaf (G17a) in comparison with standard compounds using two different elution gradients as well as full and selected MRM detection. (A) elution gradient 1, MRM detection of all targeted compounds (only those for luteolin and apigenin are shown), injection volume: 2 μL. (B) elution gradient 2, MRM detection using only the transitions for luteolin and apigenin, injection volume: 4 μL. (C) Proposed fragmentation of the [M – H]^−^ ions of luteolin and apigenin.

In addition to chlorophyll degradation products, by targeted HPLC‐MS/MS, small amounts (< 1 ng mg^−1^) of the flavonoid pigments apigenin and luteolin were detected in Geiseltal leaves and the lignite sediment matrix (Figure 5, Table S2). Retention times and MRM transitions of fossil compounds corresponded to those of the standards using two different chromatographic systems (Figure 5). The flavone apigenin can also be clearly distinguished from its isomer, the isoflavone genistein, which has a different retention time and fragmentation (Table S1). Whereas several flavonoid glycosides were detected in the 10‐year‐old freeze‐dried *Fagus* leaf (Figure S6), no flavonoid glycosides were found in the fossil leaves.

### 3.4 Carotenoid Pigments

By targeted HPLC‐MS/MS, the carotenoids lutein/zeaxanthin and β‐carotene were found in the fresh modern and the 10‐year‐old freeze‐dried *Fagus* leaf, but could not be detected in the Geiseltal leaves or sediment matrix.

## 4 Discussion

### 4.1 Exceptional Preservation of Fossil Angiosperm Metabolites

Green and brown colored leaves are found on the same sediment layers (Figure 1A), indicating differences in the composition of leaves at the time of deposition. Although higher amounts of the green CPP518 were predominantly found in green leaves, plant metabolites and their degradation products were observed in both the leaves and the sediment matrix (Table S2). The latter occurrence is due to the fact that the brown coal macerals consist also of plant material. The preservation of almost intact plant metabolites that still contain functional groups, including both primary metabolites such as chlorophyll derivatives and secondary metabolites such as polyphenolic pigments, within the about 45‐Ma‐old fossil remains of angiosperms is outstanding and goes far beyond the preservation of general plant constituents such as cutin, waxes, and lipids or their polymers observed in compression fossils (Collinson 2011; Locatelli 2014). Early degradation products co‐occurring with almost intact metabolites provide further insights into the fate of phytochemical constituents as detailed in the following paragraphs.

### 4.2 Chlorophyll and Its Degradation

**FIGURE 6:**
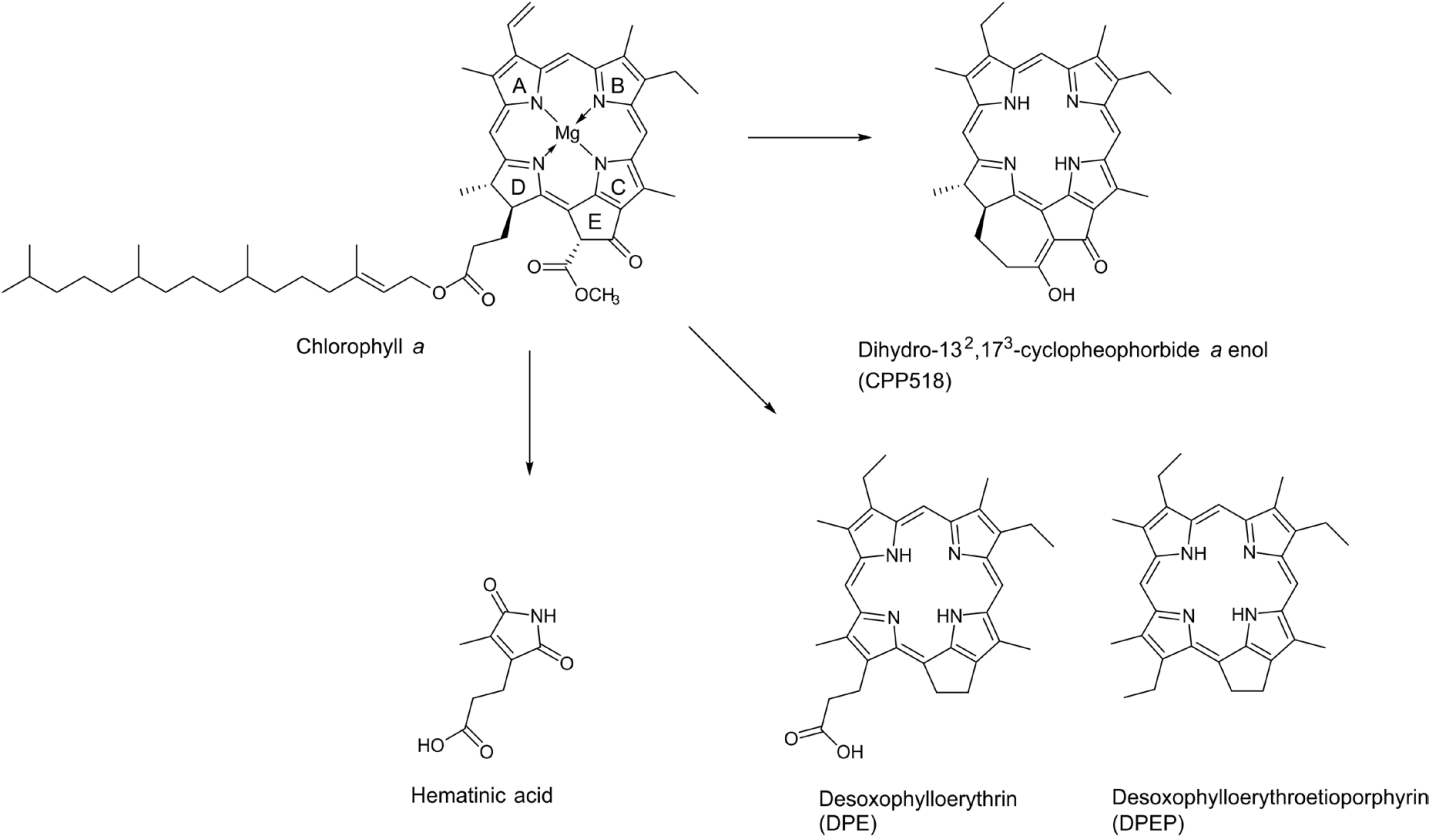
Proposed formation of diverse chlorophyll *a* degradation products in fossil leaves and lignite matrix from the Eocene of Geiseltal via different reaction pathways.

Hematinic acid is known as a chlorophyll breakdown product from present‐day senescent leaves of higher plants (Suzuki and Shioi 1999) and from in vitro degradation of chlorophyll *a* (Ritter et al. 2020), but has not yet been reported from the fossil record. Structurally related maleimides (1*H*‐pyrrole‐2,5‐diones) without a carboxyl group (Figure S2) have been found both in ancient sediments such as the Permian Kupferschiefer (Grice et al. 1996) and in recent sediments (Naeher et al. 2013). However, although maleimides are known from many sediments, their formation is still not fully understood. Methylethyl maleimide (Figure S2), which typically is the predominant maleimide, is generally thought to be derived from chlorophylls, whereas maleimides with other specific substitutions are thought to be derived from bacteriochlorophylls (Grice et al. 1996; Naeher et al. 2013). In the case of the Geiseltal leaves, a formation of hematinic acid analogous to that known from present‐day leaves is most likely. In higher plants, phyllobilins, catabolic breakdown products of chlorophylls, are formed during senescence of leaves (Kräutler 2016). These linear tetrapyrrols are rather unstable compared to the stable macrocycle of chlorophylls and can be further converted to diverse di‐ and finally monopyrrolic products such as hematinic acid (Ritter et al. 2020). Because both hematinic acid and methylethyl maleimide are found as chlorophyll breakdown products in present‐day senescent leaves (Suzuki and Shioi 1999), we propose that maleimides in modern and ancient sediments may not be formed directly from cyclic tetrapyrroles such as chlorophylls, but from linear tetrapyrroles enzymatically formed from their cyclic precursors. Hematinic acid in the Geiseltal leaves must have been formed containing the D ring of chlorophyll *a*, likely at an early stage of chlorophyll degradation before closure of the seven‐membered ring (Figure 6). This early formation is also supported by the occurrence of hematinic acid in the 10‐year‐old freeze‐dried *Fagus* leaf.

Chlorophyll‐derived pigments containing the intact core chromatophore of chlorophylls (chlorins) are commonly found in recent sediments (Callot and Ocampo 2000). Of particular relevance is the green cyclopheophorbide *a*‐enol CPP516, representing the CPP518 derivative with intact vinyl group (Figure S2). It is ubiquitous in aquatic environments, and it is often the major chlorophyll derivative in marine and lacustrine sediments (Goericke et al. 2000; Kashiyama et al. 2012). However, in contrast to modern sediments, only very few occurrences of chlorins from pre‐Quaternary sediments have been reported. Apart from methyl pheophorbide *a* described from the lignites of Geiseltal (Dilcher et al. 1970), a bicycloalkanochlorin (Figure S2) was found in addition to its porphyrin analogue in Pliocene lacustrine sediments (Keely et al. 1994), and the CPP518 degradation product dihydrochlorophyllone *a* has been reported from Late Miocene (Mawson and Keely 2008) and from Cretaceous sediments (Junium et al. 2011). Co‐occurring with CPP516, CPP518 was previously known from recent sediments (Goericke et al. 2000). However, to our knowledge, the finding of CPP518 in the Eocene Geiseltal leaves represents an unprecedented record of an intact cyclopheophorbide *a*‐enol from a fossil plant and the geological record in general.

At first sight it may be surprising to find a chlorophyll derivative preserved in fossil leaves and the lignite matrix that is known to be unstable in solution. However, cyclopheophorbide *a*‐enols are stable as a solid (Falk et al. 1975), which is also documented by their widespread occurrence in recent sediments (Goericke et al. 2000; Kashiyama et al. 2012). The instability of CPP518 in solution may also explain why this pigment was not detected in previous studies of the chlorophyll derivatives of the Geiseltal brown coal (Dilcher 1967; Dilcher et al. 1970). Accordingly, it cannot be ruled out that the CPP518 degradation product dihydrochlorophyllone *a* observed previously in extracts of Miocene and Cretaceous sediments (Mawson and Keely 2008; Junium et al. 2011) was at least in part formed artificially from CPP518 during analysis. It has been argued that the experimentally observed enol conversion to a 1:1 mixture of the (13^2^ *R*) and (13^2^ *S*) diastereomers compared to an excess of the (13^2^ *S*) diastereomer detected in natural samples would indicate an enzymatic formation of chlorophyllones (Aydin et al. 2003). However, diastereomers have different chemical properties and may thus have different stabilities in solution. Our observations show that the ratio of the two diastereomers of dihydrochlorophyllone *a* in the first measurement of an extract (Figure S3, Upper) is close to a 1:1 mixture, while in the second measurement of the same sample (Figure S3, Lower), only a few hours later, the (13^2^ *S*) diastereomer has relatively increased. In later HPLC‐DAD‐HRMS measurements of extracts, only the (13^2^ *S*) diastereomer of dihydrochlorophyllone *a* is observed (Figure S4). Despite precautionary measures for analysis of the Geiseltal samples, partial loss of CPP518 during extraction and sample preparation cannot be excluded, and it is likely that original amounts of the compound in the samples were higher than those detected. Although no standard of CPP518 was available, its concentrations in the fossil material are estimated to be in a similar range to those found for hematinic acid (based on comparison with standards of other chlorophyll derivatives and considering that the limit of detection for hematinic acid is about two orders of magnitude higher than those of the investigated chlorophyll derivatives).

Concerning the further chlorophyll derivatives pheophytin *a* and methyl pheophorbide *a* that have been reported in previous studies of the Geiseltal lignites (Dilcher 1967; Dilcher et al. 1970), it is not clear why these compounds, especially methyl pheophorbide *a*, were not found in our analyses of Geiseltal leaf samples while detectable with high sensitivity in a 10‐year‐old freeze‐dried *Fagus* leaf. One possibility is that previous samples were not collected at the same place within the Neumark‐Süd area of the Geiseltal brown‐coal pit and that local preservation conditions thus were not the same.

How was the original chlorophyll *a* in the leaves from Geiseltal converted to the cyclopheophorbide *a*‐enol CPP516 and finally to its dihydro derivative CPP518? A catabolic formation of CPP516 by herbivorous protozoans in aquatic environments has been supposed and demonstrated by feeding experiments (Goericke et al. 2000; Kashiyama et al. 2012). For details of the catabolic cyclization pathway of CPP516 see the recent study of Kashiyama et al. (2025). It has been shown that the purpose of this transformation is detoxification of fluorescent chlorophylls to non‐fluorescent cyclopheophorbide‐enols (Kashiyama et al. 2012). However, such catabolic transformation by herbivorous protozoans in situ within the leaves of Geiseltal appears rather far‐fetched. Furthermore, a cyclization of pheophorbides under natural conditions might be conceivable analogous to chemical synthesis of cyclopheophorbide *a*‐enols via a base‐catalyzed Dieckmann‐condensation (Falk et al. 1975). A diagenetic Dieckmann‐like cyclization of pyropheophorbide *a* (Figure S2) to CPP516 has been proposed for a contemporaneous marl ecosystem (Louda et al. 2000). However, such cyclization would be chemically more plausible with pyropheophorbide *a* methyl ester as the educt (Falk et al. 1975). Since no pyropheophorbide *a* and no methyl esters of pheophorbides or other chlorophyll derivatives could be detected in the Geiseltal samples, currently, the question of the direct precursor of CPP516 and thus CPP518 must remain open. Nevertheless, since cyclopheophorbide‐enols are known from recent sediments, it appears most likely that CPP518 in the Geiseltal leaves and lignites is an authentic biomarker derived from chlorophyll *a* via an early diagenetic pathway (Figure 6). This would imply that in addition to microorganisms, leaf litter may also be an important source for cyclopheophorbide‐enols in recent and ancient aquatic environments and sediments. It is assumed that the conversion from CPP516 to CPP518 (reduction of the vinyl group to ethyl) occurred at an early diagenetic stage too, since even in highly immature ancient sediments the vinyl group of cyclic tetrapyrroles has not been preserved but has been reduced to ethyl (Keely et al. 1994). In degradation experiments performed under sulfate reducing conditions, it has been shown that the reduction of the C‐3 vinyl substituent of chlorophyll *a* is a process involving anaerobic bacterial communities (Spooner et al. 1995). It has also been shown that hydrogen sulfide formed by bacterial sulfate reduction can reduce double bonds in biomolecules (Hebting et al. 2006).

The uncomplexed (geo)porphyrins DPE and DPEP have been formed diagenetically from chlorin (dihydroporphyrin) precursors following aromatization and have been previously described from highly immature ancient sediments (Keely et al. 1994). Although the Geiseltal brown coal Lagerstätte has been the subject of research for many decades, we could not find previous reports in the literature that characterized specific porphyrins from Geiseltal. Geoporphyrins are generally known from coals and sediments; however, the amounts of porphyrins in the Geiseltal samples cannot be explained only by remains from the sediment matrix from sampling of the leaves (Table S2) and must originate at least in part from the leaves themselves. Since porphyrins (and other chlorophyll derivatives), in contrast to non‐fluorescent cyclopheophorbide‐enols, are well known for their distinct red fluorescence, they may contribute to the red fluorescence that can be observed in some colored leaves from Geiseltal (Wolkenstein and Arp 2021).

### 4.3 Flavonoid Pigments and Their Degradation

Flavonoids are polyphenolic secondary metabolites that have important functions in plants and show broad biological activities including antimicrobial properties (Harborne and Baxter 1999). Flavonoid pigments typically occur in living plants in their glycosidic form (Harborne and Baxter 1999), as can be seen in the modern *Fagus* leaf (Figure S6). Since flavonoid glycosides (apart from *C*‐glycosides such as apigenin‐8*C*‐glucoside) are sensitive to hydrolysis, and from the tested flavonoids only the flavonoid aglycones apigenin and luteolin were detected at Geiseltal, it is likely that original flavonoid glycosides were diagenetically transformed into their corresponding aglycones. Apigenin‐ and luteolin‐based flavonoids are much less widespread among extant dicotyledons than the corresponding flavonols kaempferol and quercetin (Figure S2), but are common to some families such as the Lauraceae (Giannasi 1988). Members of the Lauraceae are also well represented in the Geiseltal flora (Rüffle 1976). However, since it is generally very difficult to assign isolated elongated and smooth‐edged leaves to specific families on the basis of morphological traits, it was not possible to assign the colored leaves examined in the present study to specific taxa. Although it cannot be excluded that the flavones apigenin and luteolin are diagenetic products of the flavonols kaempferol and quercetin (Figure S2), formed by the loss of one hydroxy group, this seems less likely, since the Geiseltal lignites are thermally immature and experienced no temperatures > 100°C (vitrinite reflectance VR_0_ value of 0.16%–0.33%) (Schmitz et al. 2001; Lönartz et al. 2023), which would be required for such thermolytic reactions of flavonoids (Niklas and Giannasi 1977b).

The flavonoid pigments from the Eocene of Geiseltal are significantly older than almost all previous occurrences of this class of secondary metabolites. Flavonoid aglycones and glycosides have previously been identified by paper chromatography and UV–visible spectroscopy in the Miocene Succor Creek Flora (Oregon, USA) (Giannasi and Niklas 1977; Niklas and Giannasi 1977a) and the Miocene Clarkia Flora (Idaho, USA) (Rieseberg and Soltis 1987; Niklas et al. 1985). The oldest reported flavonoids are biflavonoids from *Ginkgo* leaves from the Cretaceous of China (Huolinhe, Inner Mongolia) (Zhao et al. 2006).

### 4.4 Conditions for Preservation of Fossil Plant Metabolites

In the Geiseltal fossil Lagerstätte, rapid deposition, an anaerobic environment, and alkaline karstic water (Weigelt and Noack 1932; Krumbiegel 1975), as well as low temperatures during fossilization (Schmitz et al. 2001) enabled the exceptional chemical preservation of plant metabolites. If one compares Geiseltal with other sites that have yielded plant fossils with exceptionally well‐preserved metabolites, it is remarkable how much sedimentological conditions differ despite general similarities such as anaerobic conditions within a lacustrine paleoenvironment. Whereas *Ginkgo* leaves from Huolinhe are deposited in coal‐bearing sediments (Zhao et al. 2006), the Succor Creek Flora is preserved in very fine‐grained volcanic ash depositions (Niklas and Giannasi 1977a), the Clarkia Flora is preserved in clay sediments (Smiley and Rember 1985), and the Willershausen Flora is preserved in a laminated carbonate horizon within a succession of clay sediments (Wolkenstein and Arp 2021). More research is needed to understand in detail the factors that lead to exceptional chemical preservation of plant metabolites.

## 5 Conclusions

Screening of fossil plant extracts by targeted HPLC‐MS/MS provides a rapid overview of the quantitative composition of a wide range of intact or almost intact metabolites such as chlorophyll derivatives and polyphenolic compounds as well as their products preserved within individual specimens. Our results show the deep time stability of “unstable” cyclopheophorbide‐enols, which were found to account for the greenish color of about 45‐Ma‐old angiosperm leaves. Cyclopheophorbide‐enols are likely much more widespread in the fossil (and present‐day) record, but have probably been missed because of their sensitivity during analytical handling. Furthermore, the detection of the monopyrrolic breakdown product hematinic acid shows that widespread maleimide biomarkers in modern and ancient sediments may not be formed directly from cyclic tetrapyrroles such as chlorophylls, but from linear tetrapyrroles enzymatically formed from chlorophylls. Because of the high sensitivity and selectivity of HPLC‐MS/MS, this methodology complemented by high‐resolution MS measurements can be applied to many other fossils with preservation of organic material. Different from bulk analyses of sediments, chemical profiles of individual fossils can be directly related to their source organisms, providing insights into specific biosynthetic pathways. Such paleometabolomic‐like studies probably will provide new insights into the diversity of ancient plant metabolites, which potentially may be of chemosystematic utility. Moreover, such chemical profiles provide important information about the fossilization processes of macroorganisms at the molecular level as well as the limits of biomarker preservation.

## Author Contributions

K.W., C.E.M., V.E.M., and C.T.G. designed research; K.W., M.E., and H.F. performed research; C.E.M., M.E., and H.F. contributed new reagents or analytical tools; K.W., M.E., and H.F. analyzed data; and K.W. wrote the paper with contributions from all authors.

## Funding

This work was funded by the Deutsche Forschungsgemeinschaft (DFG) (GE 751/8, MU 1665/8‐2, MU 1665/11‐1, and EN 711/3‐1 within the Research Unit FOR 2685, as well as WO 1491/4‐2 and WO 1491/5‐1). C.E.M. is also grateful to the DFG for funding of a Sciex QTRAP 6500+ hybrid triple quadrupole‐linear ion trap mass spectrometer (INST 217/1058‐1 FUGG).

## Conflicts of Interest

The authors declare no conflicts of interest.

## Supporting information


**FIGURE S1:** Examples of dicotyledon leaf specimens from the Eocene of Geiseltal that were analyzed (indicated sample areas) in the present study. (A) Green leaf MB.Pb.1989.0200 (G2a). (B) Yellowish leaf MB.Pb.2023.0103 (G17a). (C) Yellowish leaf MB.Pb.2023.0101 (G15a) and sediment matrix (G15M). (D) Brown leaf MB.Pb.2023.0104 (G18a). Samples G2a and G17a were collected from counter slab. Scale bar, 1 cm.
**FIGURE S2:** Chemical structures of plant metabolites and their products discussed in the present work.
**FIGURE S3:** Degradation of CPP518 and formation of (*S*/*R*)‐dihydrochlorophyllone *a* from CPP518 in acetone/methanol 1:1 extract of fossil leaf (G15a) during HPLC‐MS/MS analysis (elution gradient 1, positive‐ion mode), first MRM measurement (Upper) and second MRM measurement (Lower), about 3½ h after first measurement.
**FIGURE S4:** Analytical data of dihydrochlorophyllone *a* (G2a acetone/methanol 1:1 extract, elution gradient 1). (A) HPLC chromatogram (absorbance at 395 nm) and corresponding extracted ion chromatogram (*m*/*z* 535.27, positive‐ion ESI‐HRMS). (B) UV‐visible (DAD) spectrum of peak with retention time 13.1 min shown in A. (C) Comparison of the mass spectrum of the peak in the extracted ion chromatogram shown in A (Upper) to the simulated mass spectrum for [C_33_H_34_N_4_O_3_ + H]^+^ (Lower). Note that within few days the first peak of the two dihydrochlorophyllone *a* isomers is predominant. (D) Collision‐induced fragmentation of the *m*/*z* 535.27 ion peak in the extracted ion chromatogram and proposed fragmentation of the [M + H]^+^ ion of dihydrochlorophyllone *a*.
**FIGURE S5:** MRM chromatograms (elution gradient 2, negative‐ion mode) of hematinic acid (acetone/methanol 1:1 extracts) of Geiseltal leaf (G17a) in comparison to standard compound and proposed fragmentation of the [M – H]^–^ ion of hematinic acid.
**FIGURE S6:** MRM chromatograms (quantifier, elution gradient 1, negative‐ion mode) of flavonoid pigments (acetone/methanol 1:1 extract) of 10‐year‐old freeze‐dried modern *Fagus* leaf.
**TABLE S1:** Optimized MRM transitions, collision energies (CE), and further information on compounds investigated by HPLC‐MS/MS.
**TABLE S2:** Quantitative results of leaf pigment analysis by HPLC‐MS/MS (MRM transition, quantifier).

## Data Availability

The data that support the findings of this study are available in the Supporting Information of this article.
