# Supplementary material for: Phytochemical Screening of 45‐Million‐Year‐Old Colored Angiosperm Leaves Reveals Distinctive Chlorophyll‐Derived and Polyphenolic Pigments
Source: Geobiology. 2026 Jan 31;24(1):e70042. doi: 10.1111/gbi.70042 (PMC12860425; doi:10.1111/gbi.70042)
Supplement: Supplementary file 1 — FIGURE S1: Examples of dicotyledon leaf specimens from the Eocene of Geiseltal that were analyzed (indicated sample areas) in the present study. (A) Green leaf MB.Pb.1989.0200 (G2a). (B) Yellowish leaf MB.Pb.2023.0103 (G17a). (C) Yellowish leaf MB.Pb.2023.0101 (G15a) and sediment matrix (G15M). (D) Brown leaf MB.Pb.2023.0104 (G18a). Samples G2a and G17a were collected from counter slab. Scale bar, 1 cm. FIGURE S2: Chemical structures of plant metabolites and their products discussed in the present work. FIGURE S3: Degradation of CPP518 and formation of (S/R)‐dihydrochlorophyllone a from CPP518 in acetone/methanol 1:1 extract of fossil leaf (G15a) during HPLC‐MS/MS analysis (elution gradient 1, positive‐ion mode), first MRM measurement (Upper) and second MRM measurement (Lower), about 3½ h after first measurement. FIGURE S4: Analytical data of dihydrochlorophyllone a (G2a acetone/methanol 1:1 extract, elution gradient 1). (A) HPLC chromatogram (absorbance at 395 nm) and corresponding extracted ion chromatogram (m/z 535.27, positive‐ion ESI‐HRMS). (B) UV‐visible (DAD) spectrum of peak with retention time 13.1 min shown in A. (C) Comparison of the mass spectrum of the peak in the extracted ion chromatogram shown in A (Upper) to the simulated mass spectrum for [C33H34N4O3 + H]+ (Lower). Note that within few days the first peak of the two dihydrochlorophyllone a isomers is predominant. (D) Collision‐induced fragmentation of the m/z 535.27 ion peak in the extracted ion chromatogram and proposed fragmentation of the [M + H]+ ion of dihydrochlorophyllone a. FIGURE S5: MRM chromatograms (elution gradient 2, negative‐ion mode) of hematinic acid (acetone/methanol 1:1 extracts) of Geiseltal leaf (G17a) in comparison to standard compound and proposed fragmentation of the [M – H]– ion of hematinic acid. FIGURE S6: MRM chromatograms (quantifier, elution gradient 1, negative‐ion mode) of flavonoid pigments (acetone/methanol 1:1 extract) of 10‐year‐old freeze‐dried modern Fagus leaf [file GBI-24-e70042-s001.pdf]

Supporting Information

Phytochemical Screening of 45-Million-Year-Old Colored Angiosperm Leaves Reveals Distinctive Chlorophyll-Derived and Polyphenolic Pigments

Klaus Wolkenstein[1,2], Christa E. Müller[1], Marianne Engeser[3], Holm Frauendorf[4], Victoria E. McCoy[5], Carole T. Gee[6]

[1]Pharmaceutical Institute, Pharmaceutical & Medicinal Chemistry, University of Bonn, An der Immenburg 4, 53121 Bonn, Germany

[2]Department of Geobiology, Geoscience Centre, University of Göttingen, Goldschmidtstraße 3, 37077 Göttingen, Germany

[3]Kekulé Institute for Organic Chemistry and Biochemistry, University of Bonn, Gerhard-Domagk-Str. 1, 53121 Bonn, Germany

[4]Institute of Organic and Biomolecular Chemistry, University of Göttingen, Tammannstr. 2, 37077 Göttingen, Germany

[5]Department of Geosciences, University of Wisconsin–Milwaukee, 3209 N. Maryland Avenue, Milwaukee, Wisconsin 53211, USA

[6]Bonn Institute of Organismic Biology, Division of Paleontology, University of Bonn, Nussallee 8, 53115 Bonn, Germany

Correspondence: Klaus Wolkenstein (klaus.wolkenstein@uni-goettingen.de)

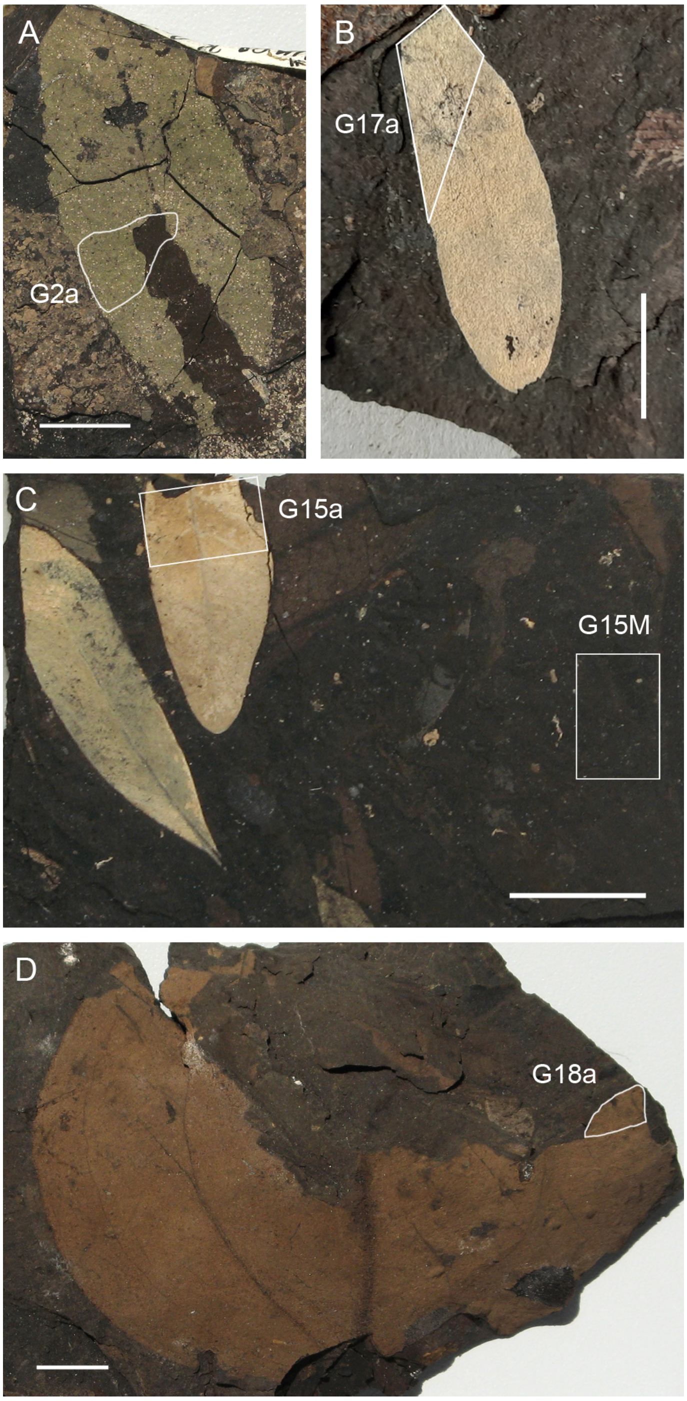

FIGURE S1 | Examples of dicotyledon leaf specimens from the Eocene of Geiseltal that were analyzed (indicated sample areas) in the present study. (A) Green leaf MB.Pb.1989.0200 (G2a). (B) Yellowish leaf MB.Pb.2023.0103 (G17a). (C) Yellowish leaf MB.Pb.2023.0101 (G15a) and sediment matrix (G15M). (D) Brown leaf MB.Pb.2023.0104 (G18a). Samples G2a and G17a were collected from counter slab. Scale bar, 1 cm.

Tetrapyrrols

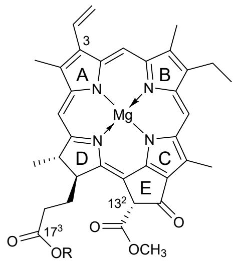

Chlorophyll *a*: R = Phytyl

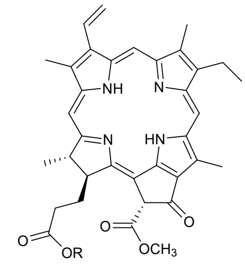

Pheophytin *a*: R = Phytyl
Methyl pheophorbide *a*: R = CH_3

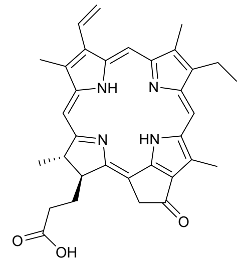

Pyropheophorbide *a*

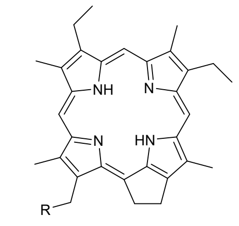

Desoxophylloerythrin
(DPE): R = CH_2-COOH
Desoxophylloerythroetioporphyrin
(DPEP): R = CH_3

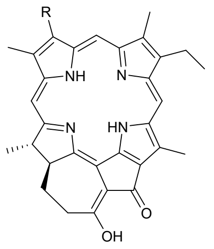

$13^2,17^3$-Cyclopheophorbide *a* enol
(CPP516): R = CH=CH_2
Dihydro-$13^2,17^3$-cyclopheo-
phorbide *a* enol
(CPP518): R = CH_2-CH_3

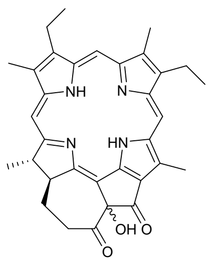

(13^2*S*/*R*)-Dihydrochlorophyllone *a*

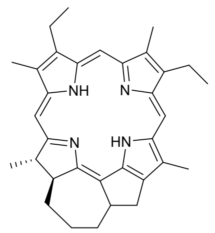

Bicycloalkanochlorin

Monopyrrols

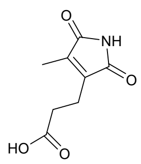

Hematinic acid

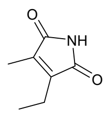

3-Ethyl-4-methyl-1*H*-pyrrole-2,5-dione
(Me,Et maleimide)

Flavonoids

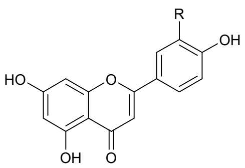

Apigenin: R = H
Luteolin: R = OH

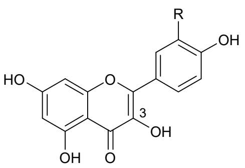

Kaempferol: R = H
Quercetin: R = OH

FIGURE S2 | Chemical structures of plant metabolites and their products discussed in the present work.

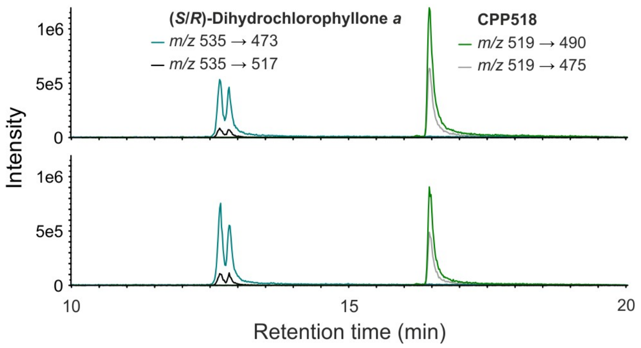

FIGURE S3 | Degradation of CPP518 and formation of (*S*/*R*)-dihydrochlorophyllone *a* from CPP518 in acetone/methanol 1:1 extract of fossil leaf (G15a) during HPLC-MS/MS analysis (elution gradient 1, positive-ion mode), first MRM measurement (Upper) and second MRM measurement (Lower), about 3½ h after first measurement.

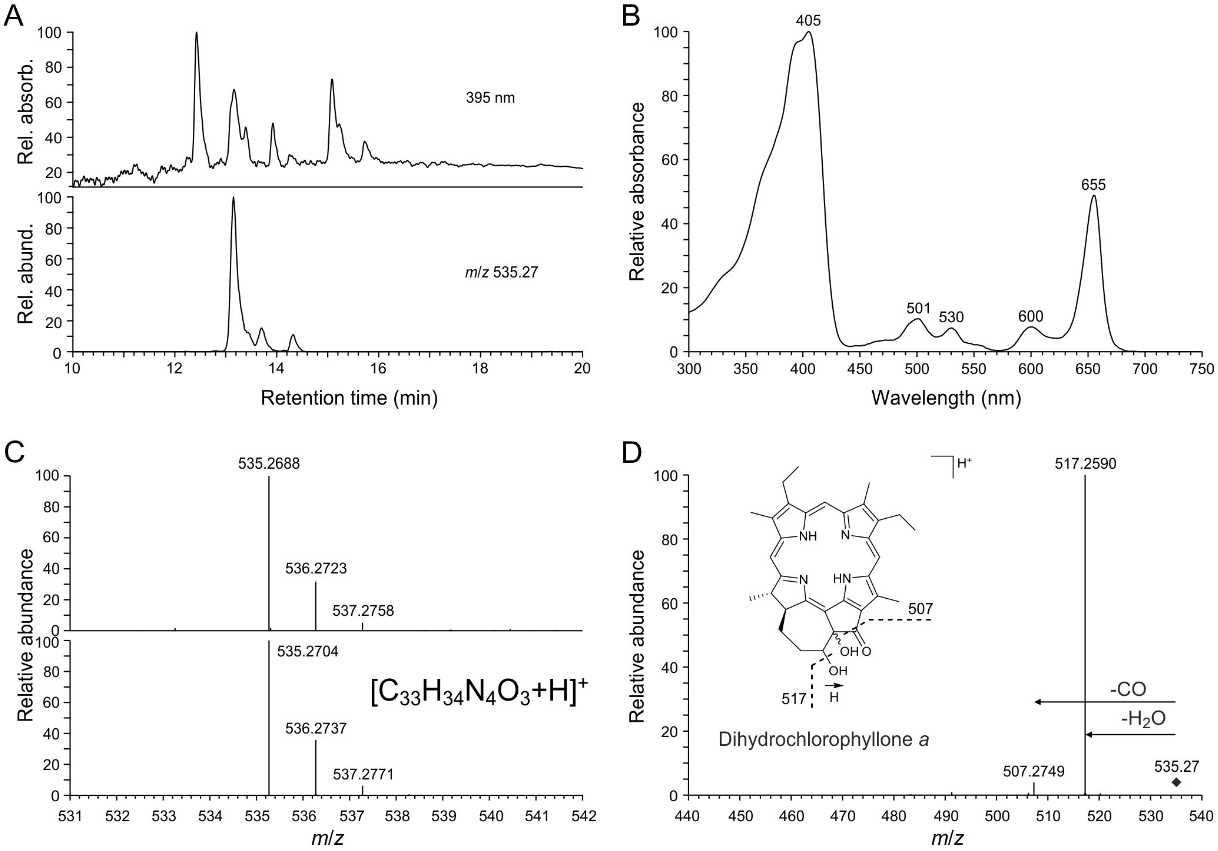

FIGURE S4 | Analytical data of dihydrochlorophyllone *a* (G2a acetone/methanol 1:1 extract, elution gradient 1). (A) HPLC chromatogram (absorbance at 395 nm) and corresponding extracted ion chromatogram (*m*/*z* 535.27, positive-ion ESI-HRMS). (B) UV-visible (DAD) spectrum of peak with retention time 13.1 min shown in A. (C) Comparison of the mass spectrum of the peak in the extracted ion chromatogram shown in A (Upper) to the simulated mass spectrum for $[C_{33}H_{34}N_4O_3+H]^+$ (Lower). Note that within few days the first peak of the two dihydrochlorophyllone *a* isomers is predominant. (D) Collision-induced fragmentation of the *m*/*z* 535.27 ion peak in the extracted ion chromatogram and proposed fragmentation of the $[M+H]^+$ ion of dihydrochlorophyllone *a*.

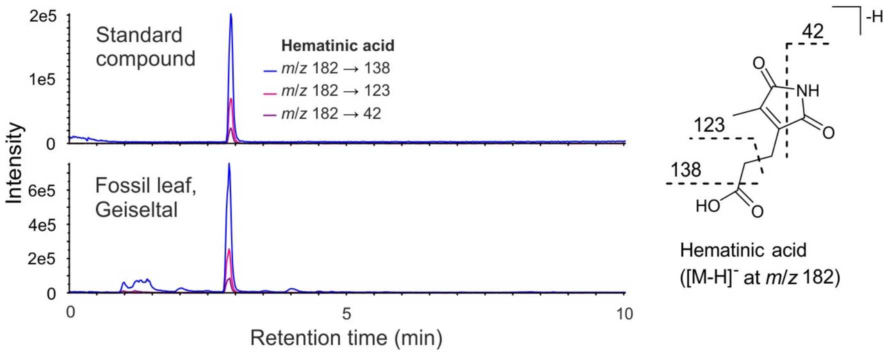

FIGURE S5 | MRM chromatograms (elution gradient 2, negative-ion mode) of hematinic acid (acetone/methanol 1:1 extracts) of Geiseltal leaf (G17a) in comparison to standard compound and proposed fragmentation of the $[M–H]^{-}$ ion of hematinic acid.

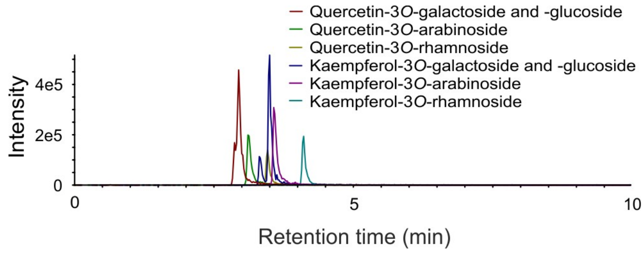

FIGURE S6 | MRM chromatograms (quantifier, elution gradient 1, negative-ion mode) of flavonoid pigments (acetone/methanol 1:1 extract) of 10-year-old freeze-dried modern *Fagus* leaf.

TABLE S1 | Optimized MRM transitions, collision energies (CE), and further information on compounds investigated by HPLC-MS/MS.

Retention time (min)[a]	Compound	Molecular formula	Molecular or quasimolecular ion	MRM transition, quantifier, CE (V)	MRM transition, qualifier 1, CE (V)	MRM transition, qualifier 2, CE (V)
1.17	Hematinic acid[b]	$C_8H_9NO_4$	$[M–H]^-$	182 → 138, 12	182 → 123, 26	182 → 42, 60
2.25	*p*-Coumaric acid[b]	$C_9H_8O_3$	$[M–H]^-$	163 → 93, 42	163 → 117, 42	
2.70	Apigenin-8*C*-glucoside[b]	$C_{21}H_{20}O_{10}$	$[M–H]^-$	431 → 311, 30	431 → 283, 46	
2.84	Quercetin-3*O*-rutinoside[b]	$C_{27}H_{30}O_{16}$	$[M–H]^-$	609 → 300, 48	609 → 271, 70	
2.90	Quercetin-3*O*-galactoside[b]	$C_{21}H_{20}O_{12}$	$[M–H]^-$	463 → 300, 36	463 → 271, 56	
2.95	Quercetin-3*O*-glucoside[b]	$C_{21}H_{20}O_{12}$	$[M–H]^-$	463 → 300, 36	463 → 271, 54	
3.00	Dihydrokaempferol[b]	$C_{15}H_{12}O_6$	$[M–H]^-$	287 → 259, 18	287 → 125, 28	
3.12	Quercetin-3*O*-arabinoside[c]	$C_{20}H_{18}O_{11}$	$[M–H]^-$	433 → 300, 36	433 → 271, 54	
3.33	Kaempferol-3*O*-galactoside[c]	$C_{21}H_{20}O_{11}$	$[M–H]^-$	447 → 284, 38	447 → 255, 52	
3.48	Quercetin-3*O*-rhamnoside[b]	$C_{21}H_{20}O_{11}$	$[M–H]^-$	447 → 300, 34	447 → 271, 54	
3.51	Kaempferol-3*O*-glucoside[b]	$C_{21}H_{20}O_{11}$	$[M–H]^-$	447 → 284, 38	447 → 255, 52	
3.58	Kaempferol-3*O*-arabinoside[c]	$C_{20}H_{18}O_{10}$	$[M–H]^-$	417 → 284, 38	417 → 255, 52	
4.10	Kaempferol-3*O*-rhamnoside[c]	$C_{21}H_{20}O_{10}$	$[M–H]^-$	431 → 284, 38	431 → 255, 52	
4.66	Quercetin[b]	$C_{15}H_{10}O_7$	$[M–H]^-$	301 → 151, 28	301 → 179, 24	
5.00	Luteolin[b]	$C_{15}H_{10}O_6$	$[M–H]^-$	285 → 133, 42	285 → 151, 34	285 → 107, 36
5.02	Genistein[b]	$C_{15}H_{10}O_5$	$[M–H]^-$	269 → 133, 40	269 → 159, 40	
5.65	Kaempferol[b]	$C_{15}H_{10}O_6$	$[M–H]^-$	285 → 185, 36	285 → 211, 40	

5.77	Apigenin[b]	$C_{15}H_{10}O_5$	$[M–H]^–$	269 → 117, 42	269 → 151, 32	269 → 107, 36
12.30	Desoxophylloerythrin[d]	$C_{33}H_{36}N_4O_2$	$[M+H]^+$	521 → 447, 61	521 → 433, 63	
12.63	(*S*)-Dihydrochlorophyllone *a*[d]	$C_{33}H_{34}N_4O_3$	$[M+H]^+$	535 → 473, 63	535 → 517, 41	
12.76	Pheophorbide *a*[b]	$C_{35}H_{36}N_4O_5$	$[M+H]^+$	593 → 533, 49	593 → 460, 61	
12.81	(*R*)-Dihydrochlorophyllone *a*[d]	$C_{33}H_{34}N_4O_3$	$[M+H]^+$	535 → 473, 63	535 → 517, 41	
13.53	Methyl pheophorbide *a*[b]	$C_{36}H_{38}N_4O_5$	$[M+H]^+$	607 → 547, 49	607 → 460, 59	
14.80	DPEP[d]	$C_{32}H_{36}N_4$	$[M+H]^+$	477 → 447, 61	477 → 433, 61	
15.40	Lutein[b]	$C_{40}H_{56}O_2$	$[M]^{+\bullet}$	568 → 476, 19	568 → 551, 15	
15.40	Zeaxanthin[b]	$C_{40}H_{56}O_2$	$[M+H]^+$	569 → 477, 17	569 → 551, 15	
16.46	CPP518[d]	$C_{33}H_{34}N_4O_2$	$[M+H]^+$	519 → 490, 51	519 → 475, 65	
18.16	Chlorophyll *a*[b]	$C_{55}H_{72}MgN_4O_5$	$[M+H]^+$	893 → 615, 39	893 → 482, 75	
19.10	Pheophytin *a*[b]	$C_{55}H_{74}N_4O_5$	$[M+H]^+$	871 → 593, 51	871 → 533, 61	
19.48	β-Carotene[b]	$C_{40}H_{56}$	$[M]^{+\bullet}$	536 → 444, 19		

[a]Elution gradient 1

[b]Standards from compound library of Pharmaceutical Institute, University of Bonn

[c]Compounds not present in compound library of Pharmaceutical Institute, University of Bonn; MRM transitions derived based on observed transitions of related flavonoid glycoside standards; retention times are those observed during analysis of modern *Fagus* leaf

[d]Compounds found by non-targeted HPLC-DAD-HRMS of fossil leaf extract from Geiseltal

TABLE S2 | Quantitative results of leaf pigment analysis by HPLC-MS/MS (MRM transition, quantifier).

Sample	Collection no.	Description	Sample amount (mg)	Matrix content of sample (estimated %)	CPP518 (relative peak area per mg of sample)[a]	DPE (relative peak area per mg of sample)	Hematinic acid concentration (ng per mg of sample)[b]	Luteolin concentration (ng per mg of sample)[b]	Apigenin concentration (ng per mg of sample)[b]
G1a	MB.Pb.2023/0087.1	Green leaf	30.3	80	1.00	0.26	49.1	0.06	0.07
G2a	MB.Pb.1989/0200	Green leaf	36.9	80	0.99	0.55	50.4	0.04	0.09
G5a	MB.Pb.2023/0091	Green leaf	22.1	20	0.35	0.20	22.0	n.d.	n.d.
G5M	MB.Pb.2023/0091	Matrix of G5a	77.2	100	0.05	0.04	8.6	0.03	0.06
G7a	MB.Pb.2023/0093	Green leaf	127.7	80	0.21	0.23	17.6	0.04	0.05
G14a	MB.Pb.2023.0100	Green leaf	2.5	10	0.80	1.00	51.1	n.d.	n.d.
G15a	MB.Pb.2023.0101	Yellowish leaf	3.0	10	0.43	0.67	32.5	n.d.	n.d.
G15M	MB.Pb.2023.0101	Matrix of G15a	12.6	100	0.48	0.59	30.5	n.d.	n.d.
G17a	MB.Pb.2023.0103	Yellowish leaf	77.5	80	0.38	0.19	34.0	0.05	0.06
G18a	MB.Pb.2023.0104	Brown leaf	1.2	10	0.79	0.73	44.8	n.d.	n.d.
G22a	MB.Pb.2023.0088	Yellowish leaf	2.6	10	0.26	0.30	23.8	0.40	0.77
GP3	MB.Pb.2025/0299.2	Green leaf	6.0	10	0.70	0.48	39.0	n.d.	n.d.

[a]Because of the observed instability of CPP518 in acetone/methanol 1:1 solution, partial loss of the compound during extraction, sample preparation and measurement is expected despite precautionary measures, only the first measurement of the sample was used for quantification.

[b]Relative SD of technical replicates was <18%.

n.d., not detectable. Limits of detection (MRM): hematinic acid, 10 ng mL^{-1}; luteolin, 1 ng mL^{-1}; apigenin, 0.5 ng mL^{-1}.
